# Editorial Department

**Published:** 1842-01

**Authors:** 


### THE WESTERN JOURNAL

Vol. V.—No. I

LOUISVILLE, JANUARY 1, 1842.

### EDITORIAL DEPARTMENT.

After presenting to our readers the compliments of the season, and wishing for each his complement of holy-day enjoyments (none of course being unhallowed) with greater success in acquiring and curing patients throughout the coming than he had in the departing year; after all these gratuitous expressions of kindness to them, we beg leave to add for their further gratification, that, by our request, the publishers of the Journal have engaged, as assistant and junior editor, our young friend, T. W. Colescott, M. D., already advantageously known to our readers, as a translator from the French of some of the valuable foreign articles published by us. It will be the special duty of Dr. C. to edit (we had almost said correct) and fit for publication, such MSS. as have been thrown off by their authors so hastily, as to require some modification. He will likewise be charged with the supervision of the press, and with the preparation of such analytical reviews and bibliographical notices, as will supply our felt and admitted, but unavoidable deficiencies.

